# Supplementary material for: Modelling Skylarks (Alauda arvensis) to Predict Impacts of Changes in Land Management and Policy: Development and Testing of an Agent-Based Model
Source: PLoS One. 2013 Jun 6;8(6):e65803. doi: 10.1371/journal.pone.0065803 (PMC3675089; doi:10.1371/journal.pone.0065803)
Supplement: Supporting Information S4 — The skylark ODdox as a zipped archive. (ZIP) [file pone.0065803.s004.zip › Skylark_ODdox/configurator_8h.html]

ALMaSS Skylark ODdox: configurator.h File Reference


ALMaSS Skylark ODdox
 2.0


- Main Page
- Related Pages
- Classes
- Files

- File List
- File Members

Classes |
Macros |
Enumerations |
Variables

configurator.h File Reference

`#include <stdio.h>`  
`#include <string>`  
`#include <map>`  
`#include <vector>`

|  |  |
| --- | --- |
| Classes | |
| class | CfgBase |
|  | Base class for a configurator entry. More... |
| class | CfgBool |
|  | Bool configurator entry class. More... |
| class | CfgFloat |
|  | Double configurator entry class. More... |
| class | CfgInt |
|  | Integer configurator entry class. More... |
| class | CfgStr |
|  | String configurator entry class. More... |
| class | Configurator |
|  | A class to provide standard parameter entry facilities. More... |

|  |  |
| --- | --- |
| Macros | |
| #define | CFG\_MAX\_LINE\_LENGTH   512 |

|  |  |
| --- | --- |
| Enumerations | |
| enum | CfgSecureLevel { CFG\_CUSTOM, CFG\_PUBLIC, CFG\_PRIVATE } |
| enum | CfgType {     CFG\_NONE, CFG\_INT, CFG\_FLOAT, CFG\_BOOL,     CFG\_STRING   } |

|  |  |
| --- | --- |
| Variables | |
| class Configurator \* | g\_cfg |

---

## Macro Definition Documentation

#define CFG\_MAX\_LINE\_LENGTH   512

Referenced by Configurator::DumpSymbols(), Configurator::ParseCfgLine(), and Configurator::ReadSymbols().

---

## Enumeration Type Documentation

enum CfgSecureLevel

**Enumerator:**
:   |  |  |
    | --- | --- |
    | *CFG\_CUSTOM* |  |
    | *CFG\_PUBLIC* |  |
    | *CFG\_PRIVATE* |  |

{

CFG\_CUSTOM,

CFG\_PUBLIC,

CFG\_PRIVATE

} CfgSecureLevel;

enum CfgType

**Enumerator:**
:   |  |  |
    | --- | --- |
    | *CFG\_NONE* |  |
    | *CFG\_INT* |  |
    | *CFG\_FLOAT* |  |
    | *CFG\_BOOL* |  |
    | *CFG\_STRING* |  |

{

CFG\_NONE,

CFG\_INT,

CFG\_FLOAT,

CFG\_BOOL,

CFG\_STRING

} CfgType;

---

## Variable Documentation

class Configurator\* g\_cfg

Referenced by CfgBase::CfgBase(), CloseDownSim(), Landscape::DumpAllSymbolsAndExit(), Landscape::DumpPublicSymbols(), Landscape::Landscape(), and Landscape::ReadSymbols().


- CJT
- MSVC
- ALMaSS Working Source
- Landscape
- configurator.h
- Generated on Thu Jan 10 2013 13:15:35 for ALMaSS Skylark ODdox by
   1.8.1.1
